# Velopharyngeal Incompetence in Musicians: A State-of-the-Art Review

**DOI:** 10.3390/jpm13101477

**Published:** 2023-10-09

**Authors:** Lucía Mata-Pose, Miguel Mayo-Yáñez, Carlos M. Chiesa-Estomba, Jérôme R. Lechien, Luigi A. Vaira, Antonino Maniaci, Alberto M. Saibene, Christian Calvo-Henríquez, Irma Cabo-Varela

**Affiliations:** 1 School of Medicine, Universidade de Santiago de Compostela (USC), 15782 Santiago de Compostela, Spain; 2 Otorhinolaryngology—Head and Neck Surgery Department, Complexo Hospitalario Universitario A Coruña (CHUAC), 15006 A Coruña, Spain; irma.cabo.varela@sergas.es; 3 Otorhinolaryngology—Head and Neck Surgery Department, Hospital San Rafael (HSR) de A Coruña, 15006 A Coruña, Spain; 4 Young-Otolaryngologists of the International Federation of Oto-Rhino-Laryngological Societies (YO-IFOS) Study Group, 75000 Paris, France; chiesaestomba86@gmail.com (C.M.C.-E.); jerome.lechien@umons.ac.be (J.R.L.); luigi.vaira@gmail.com (L.A.V.); tnmaniaci29@gmail.com (A.M.); alberto.saibene@gmail.com (A.M.S.); christian.calvo.henriquez@gmail.com (C.C.-H.); 5 Otorhinolaryngology—Head and Neck Surgery Department, Hospital Universitario Donostia—Biodonostia Research Institute, 20014 Donostia, Spain; 6 Department of Otolaryngology, Polyclinique de Poitiers, Elsan Hospital, 86000 Poitiers, France; 7 Department of Otolaryngology—Head & Neck Surgery, Foch Hospital, School of Medicine, UFR Simone Veil, Université Versailles Saint-Quentin-en-Yvelines (Paris Saclay University), 91190 Paris, France; 8 Department of Human Anatomy and Experimental Oncology, UMONS Research Institute for Health Sciences and Technology, University of Mons (UMons), 7000 Mons, Belgium; 9 Department of Otolaryngology—Head & Neck Surgery, Centre Hospitalier Universitaire Saint-Pierre, 1000 Brussels, Belgium; 10 Maxillofacial Surgery Operative Unit, Department of Medicine, Surgery and Pharmacy, University of Sassari, 07100 Sassari, Italy; 11 Faculty of Medicine and Surgery, University of Enna “Kore”, 94100 Enna, Italy; 12 Otolaryngology Unit, Santi Paolo e Carlo Hospital, Department of Health Sciences, Università degli Studi di Milano, 20122 Milan, Italy; 13 Service of Otolaryngology, Hospital Complex of Santiago de Compostela, 15701 Santiago de Compostela, Spain; 14 Health Sciences Programme, International Center for Doctorate (EIDUDC), Universidade da Coruña (UDC), 15001 A Coruña, Spain

**Keywords:** insufficiency velopharyngeal, velopharyngeal incompetence, musicians, wind musicians, head neck

## Abstract

The velopalatine sphincter is a muscular valve that creates a hermetic seal between the nasopharynx and the oropharynx. It guarantees phonation, swallowing, and breathing (forces expirations). In wind musicians, sphincter closure must be precise during sound generation. Its failure will cause velopharyngeal incompetence (VPI) and the end of professional success. The objective of this article was to conduct a state-of-art review of VPI in wind musicians with a systematic approach based on the PRISMA Statement. The etiology, epidemiology, clinic, diagnosis, and treatment of VPI in wind musicians were evaluated. The research was carried out in different databases (PubMed/MEDLINE, the Cochrane Library, Scielo) and through the *Mergullador* metasearch engine. A total of 20 publications were selected. VPI is a pathology that affects around one-third of wind musicians according to studies. It causes pharyngeal noises and nasal air emissions during performance. The main etiology seems to be the fatigue of the velopalatine sphincter muscles. The most used diagnostic techniques consist of clinical history, physical examination, and nasofibroscopy. There is no consensus among authors about therapeutic management. Future investigations are necessary to confirm that fatigue of velopalatine sphincter muscles and other factors that increase it are the main causes of VPI in wind musicians.

## 1. Introduction

Velopharyngeal dysfunction (VPD) refers to the functional incompetence of the velopharyngeal sphincter that connects the oropharynx with the nasopharynx. Three types of VPD correspond to different etiologies. Velopharyngeal Insufficiency is commonly used to describe a functional impairment resulting from anatomical or structural causes. Velopharyngeal incompetence (VPI) refers to neurophysiological disorders that prevent complete closure of the sphincter. Lastly, velopharyngeal mislearning is a speech articulation disorder in which oral articulation phonemes are incorrectly positioned over the pharynx, resulting in abnormal sphincter behavior. Although these terms are often used interchangeably in everyday language, their theoretical differentiation holds significant clinical relevance, as different types of velopharyngeal dysfunction require distinct treatments and have varying prognoses [1].

There are numerous causes of VPI, such as velopharyngeal dysplasia, DiGeorge syndrome, oral tumors, neurological disorders, and abnormalities in Waldeyer’s ring. However, the typical and most frequent form of VPI affects pediatric patients with a cleft palate. It is primarily manifested by nasal voice, nasal air emissions during the speech, difficulties in articulating various phonemes, and in severe cases, regurgitation of food into the nasal cavity [2]. On the other hand, there is a unique group of affected patients who do not present this classical clinical picture and respond to a different causal process, wind musicians.

VPI in wind musicians was first described in 1970 by Weber and Chase in an oboist [3]. In 1979, Dibbell et al. reported two new cases of VPI in woodwind musicians that were successfully corrected using a superiorly based V-Y pharyngeal flap [4]. Over the years, the number of publications on this topic has grown as healthcare professionals and individuals in the field of music have become aware of the importance of this condition. Playing a wind instrument requires a constant flow of air at sufficient pressure to overcome the resistance to vibration offered by the instrument’s mouthpiece and produce sound. The sound emission begins with a forced expiration, where the air travels through a sealed pathway—the airways—starting from the lungs and passing through the bronchi, larynx, pharynx, and oral cavity until it reaches the interior of the instrument. To achieve this, when blowing, the soft palate contracts and, aided by intraoral air pressure, moves posteriorly and upward to close the communication between the oropharynx and the nasopharynx, preventing air from escaping through the nose. This would result in an inefficient air column and suboptimal sound quality. Specifically, it has been observed in wind musicians that the muscles particularly involved in the closure of the velopharyngeal sphincter are the tensor veli palatini and the levator veli palatini. However, the palatopharyngeus muscle along with the uvula contribute as well, adding volume to the soft palate and improving the seal. On the other hand, when the palatopharyngeus and palatoglossus muscles work together, they open the nasal cavity and elevate the posterior part of the tongue, a movement related to a continuous breathing technique known as circular breathing [5], which involves inhaling without interrupting the sound. Therefore, the proper functioning of the velopharyngeal sphincter ensures the maximum utilization of pressure and airflow for the production of high-quality sound. Additionally, it has been observed that the soft palate directly or indirectly participates in sound effects such as vibrato and may even play a fundamental role in double-tonguing techniques [6].

A malfunction of the velopharyngeal sphincter results in noticeable nasal or pharyngeal sounds for the audience or in recordings, sound without depth or direction, difficulty playing musical passages that require high air pressure (extremely high registers, fortissimo dynamics, etc.), intonation problems, and even the inability to play in severe cases [7]. Despite being a recognized issue among music professionals and healthcare professionals, there is currently no consensus within the scientific community regarding the etiology, epidemiology, clinical presentation, diagnosis, and treatment of VPI in wind musicians.

The objective of this review was to systematically evaluate the available literature and current knowledge of VPI, identify future research lines on this topic, and explore potential personalized approaches.

## 2. Materials and Methods

Although this is not a systematic review, a systematic approach for the search strategy in peer-reviewed journals based on the Preferred Reporting Items for Systematic Reviews and Meta-Analyses (PRISMA) Statements (see Supplementary Checklist) [8] was adopted. The review process was structured using a modified population, intervention, comparison, outcome (PICO) framework [9], also adapted for experimental/basic research studies in human and animal tissues.

### 2.1. Participants

Woodwind and brass wind musicians, both professionals and amateurs, were included. The authors extracted substantial information about the study population and publication (year, country, and study design), sociodemographic variables (number, age, and gender of participants), history of upper airway-digestive surgeries, type of instrument, practice hours, associated symptoms, associated pathologies, and toxic habits, among others.

### 2.2. Intervention

All studies in which researchers studied aspects (evaluation, prevalence, diagnosis, treatment) of the possible relationship between VPI and musical practice with wind instruments were evaluated.

### 2.3. Comparison

The presence of a control group or musicians practicing another instrument was assessed. The absence of a control group did not lead to exclusion from the review.

### 2.4. Outcomes

The primary outcome studied was to review the scientific evidence of a potential association between VPI and playing wind instruments. The secondary outcome was a review of basic science studies to evaluate the development, diagnosis, and treatment of VPI. Due to the heterogeneity in the definition of VPI, variants of nomenclature such as velopharyngeal insufficiency were included in order to encompass all the available scientific literature on the subject.

### 2.5. Search Strategy

During December 2022, two authors (LMP; MMY) independently conducted a search in different indexed databases (PubMed/MEDLINE, the Cochrane Library, Scielo, ScienceDirect) and through the *Mergullador* meta-search (https://bibliosaude.sergas.gal, accessed on 1 December 2022) engine using the following keywords: *((music OR musician) OR (brass OR wood OR wind)) AND (velopharyngeal insufficiency OR velopharyngeal incompetence)*.

No inclusion criteria were applied based on the publication date. All studies (human and animal) published in peer-reviewed journals that evaluated the possible relationship between VPI and playing woodwind or brass wind instruments were considered. Preprints, gray literature, and conference communications were not considered. No eligibility criteria were applied regarding the study type (both experimental and observational, prospective, and retrospective). Only studies published in Spanish, English, Italian, French, or Portuguese were considered.

**Figure 1:**
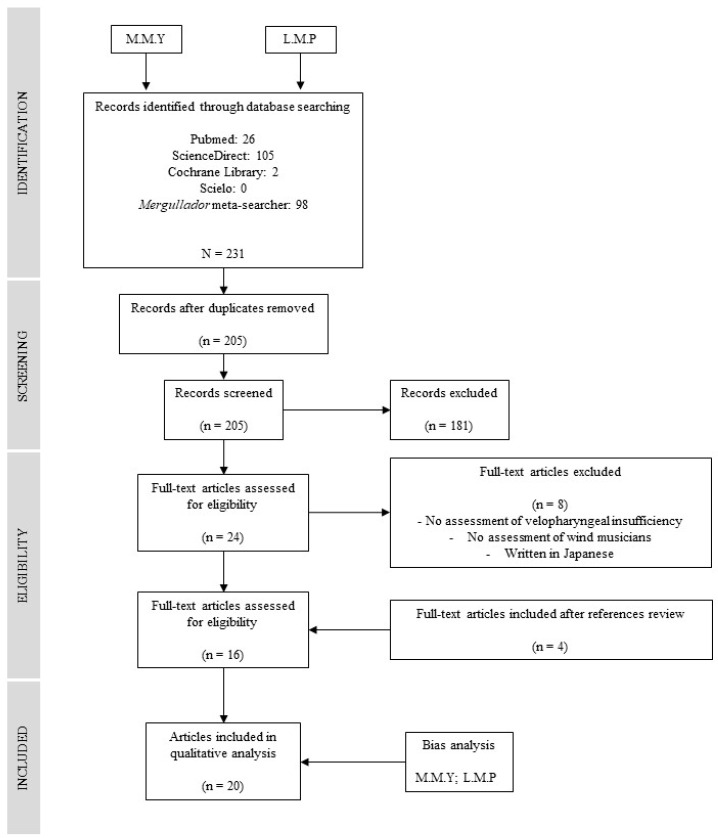
Flowchart of the systematic review according to PRISMA guidelines.

The authors examined the abstracts of publications and available full texts that referenced the technique. Once duplicates were removed and articles were selected, the authors read the full texts and bibliographic references of all manuscripts to include possible studies not found through the search strategy. Disagreements among the authors were discussed within the team, and decisions were made by consensus (Figure 1).

### 2.6. Analysis of Evidence and Biases

Data extraction was performed in duplicate to avoid errors in the qualitative analysis. For publications from the same center with the possibility of duplicate samples, they were included for qualitative analysis, and only those with larger sample sizes were included for quantitative analysis. The level of evidence was classified according to the levels of the Oxford Centre for Evidence-Based Medicine [10]. The methodological quality of the selected studies was assessed using the Public Health Guidance tool from the National Institute for Health and Care Excellence (NICE) [11].

## 3. Results

After evaluating the full text and the application of inclusion criteria, a total of 20 articles were selected. The main aspects assessed include symptomatology, personal history, risk factors, diagnostic techniques, and therapeutic management of wind musicians with VPI (Supplementary Table S1).

The majority of the publications were geographically located in the United States [3,4,12,13,14,15,16,17,18,19,20,21,22,23,24,25], with two publications from Australia [26,27] and two from Europe, one from Germany [7] and another from the Netherlands [28]. Temporally, these studies spanned 51 years—from 1970 to 2021—with only eight published in the last 10 years from the current date. Thirteen were case series [3,4,12,13,14,15,16,20,22,23,24,26,28], four were cross-sectional studies [7,17,18,27], two were case-control studies [19,21], and one was a semi-systematic review [25]. The Evans et al. working group contributed to three of the included publications [18,21,27] and collaborated with other authors, showing a special interest in this condition.

The total number of participants across all studies was 424 wind musicians from different professional and educational levels, with 30% being male and 32% female. Gender was not specified for the remaining participants [7]. Instruments were represented in descending order of frequency: 58 trumpets, 50 clarinets, 46 oboes, 43 flutes, 41 horns, 37 trombones, 28 bassoons, 27 saxophones, 16 tubas, 10 euphoniums, and 9 baritone horns. The specific type of instrument was not mentioned for the remaining participants [18]. Musicians’ ages ranged from a minimum of 12 years to a maximum of 39 years. Additionally, the opinions of 174 healthcare professionals were considered, including 84 plastic surgeons, 77 otorhinolaryngologists, and 7 speech therapists.

The available evidence was found to be of low quality. A total of 60% of the studies were classified as regular quality and the remaining 40% as poor quality (Table 1 and Supplementary Table S2).

The eight excluded publications are summarized in Supplementary Table S3. There were three cross-sectional studies [6,29,30], three non-systematic reviews [2,31,32], one case series [33], and one systematic review [34]. The main reasons these articles were not considered in the present review were the lack of participation of wind musicians in the studies [2,29,34], the absence of symptomatology or compatible diagnostic suspicion with VPI [6,30], a language different from Spanish or English [33], and topics unrelated to VPI in wind musicians [31,32].

## 4. Discussion

Playing a wind instrument requires integrating, with the sensitivity that the art of music demands, broad musical knowledge, interpretive skills, psychomotor abilities, and control of anatomical structures such as the respiratory system, fingers, lip muscles, and also the velopharyngeal mechanism. When any of these elements fail, the quality of the musical performance is affected. VPI can become a serious and debilitating issue for music professionals. Currently, there is no consensus on the medical management and rehabilitation of VPI in wind musicians. It is important to provide healthcare professionals with information about etiology, epidemiology, clinical presentation, diagnosis, and treatment so that they are aware of the specifics of this disorder. This discussion thoroughly analyzes the data obtained from the scientific literature review.

### 4.1. Concept and Nomenclature

VPI in wind musicians refers to the inability to fully close the velopharyngeal sphincter due to fatigue of the sphincteric muscles, resulting in the characteristic symptoms of pharyngeal noises and nasal air emission during musical performance. The first description of this disorder in the scientific literature was by Weber et al. in 1970, primarily based on clinical criteria and outlining possible causes [3]. Until 2007, this was the accepted definition of VPI by all authors. However, a study by Malick et al. in the same year elaborates on the etiology and professional life impact of musicians as part of the concept of VPI [17]. Other authors add to this new definition specifics such as the latency time of symptom onset or groups of patients most affected by age or professional level [18]. Nevertheless, these elements do not seem to conceptually or generically represent velopharyngeal incompetence in wind musicians due to the significant variability among samples and studies.

Different terms for the same condition have been observed in the literature: stress velopharyngeal incompetence [3,4,13,14,15,17,19,20,22,24,28], stress velopharyngeal insufficiency [7,21,27], velopharyngeal insufficiency [16,23,26,29], and velopharyngeal incompetence [12,26]. These terminological variations result in differences in etiology, treatment, and prognosis of velopalatal dysfunction [2]. However, in the case of wind musicians, this discrepancy in naming might reflect the conglomerate of causes as well as predisposing and exacerbating factors that combine in a musician experiencing velopharyngeal dysfunction [17,27]. It is also likely related to the direct translation of the English words “incompetence” and “insufficiency” to the Spanish scientific literature. Considering fatigue alone as a common and characteristic cause within this group of patients, the most accurate designation would likely be velopharyngeal incompetence [2], mostly used due to its alignment with a neuromuscular mechanism.

However, in cases where anatomical alterations, fatigue of the sphincteric musculature, and factors of other nature (psychological, related to the instrument, etc.) coexist, the use of the term “insufficiency” could be justified, a word that, furthermore, in the Spanish language has a broader meaning that describes the inability of proper organ functioning. Similarly, the diversity in the therapeutic management of VPI in wind musicians proposed in the literature, along with the different outcomes obtained, also suggests that this specific type of velopharyngeal dysfunction could be a multifactorial process that may be appropriately addressed by various terms and treatments depending on the individual circumstances of each patient. In conclusion, it appears challenging to select the perfect term to accurately describe the reality of VPI in wind musicians.

### 4.2. Presentation of VPI in Wind Musicians

The presentation of VPI in wind musicians possesses characteristics that distinguish it from other causes of velopharyngeal incompetence. The defining symptoms are nasal air emissions (NAEs) and pharyngeal noises during instrumental practice, which may occur separately or simultaneously [3]. The most frequent symptom varies based on the analyzed sample. Malick et al. describe that 53% of their patients experienced both symptoms, while 30% had only noises, and 17% had NAEs [17]. However, Evans et al. report that 83% exclusively presented pharyngeal noises. The entire reviewed literature agrees that musicians experiencing any of these manifestations suffer from VPI, except for the study by Bennet et al., which suggests that some musicians with this symptomatology also experience nasal air leaks before sound production in the context of a completely normal and functional velopharyngeal structure [19]. These findings question whether the symptoms are predictors of VPI in all cases.

The latency period of symptom onset from the start of instrumental practice is mainly after 30 min (45.3% according to Malick et al. and 43% in the study by Evans et al.) [12,14,17,18,27]. While it is true that cases have been described where symptoms appear immediately [13] or after 10 min [3], it is also important to note that in most studies the latency period is not specified, making it difficult to conclude whether there is a relationship between the time of music played and the onset of symptoms. The delay in symptom onset could be attributed to the different interindividual combinations of factors and cofactors contributing to velopharyngeal incompetence in wind musicians.

Based on the clinically based diagnostic criterion, the prevalence of VPI in wind musicians has been observed to be 31–39% [7,17,18] according to the conducted observational studies. It primarily affects oboists and clarinetists, although the frequency order varies depending on the obtained sample [27]. These variations could be due to the different distributions of musicians per instrument type in the included studies. For example, in the reviewed case series, there are reported five cases of clarinets, four oboes, three trumpets, two horns, two saxophones, one bassoon, and one trombone. Likewise, in the cross-sectional study by Schwab et al., the largest number of participants play the oboe, horn, and bassoon [7]; in Malick et al.’s study, the trumpet, flute, and saxophone [17], while Evans et al. (2011) does not specify [18]. These data could help establish a relationship with the pathophysiology of VPI; however, they must be interpreted with caution due to the limited sample size of the case series (total of 18 cases) and the observational studies working with samples that do not appear to be sufficiently representative of the musician population (ranging from 77 to 156 participants).

Another noteworthy aspect is the age of onset of VPI symptoms. Typically, it appears in young musicians between the ages of 12 and 18 during their formative years [3,12,14,16,20,24]. In the study by Schwab et al., which includes both professional orchestra musicians and students, a higher frequency of VPI is observed among students (26%) compared to veterans (15%) [7]. One possible explanation for the higher prevalence of VPI in youth is the physiological atrophy of adenoid tissue, which could compromise the proper closure of the velopharyngeal sphincter [35]. Another possible explanation is the difference in experience and expertise among professionals, with orchestra musicians being better able to control velopharyngeal closure.

Lastly, it is not clear whether the prevalence of VPI differs between males and females. In the study by Evans et al. (2011) [18], 36% of women experienced this disorder compared to 44% of men. However, Schwab et al. reported that symptoms were more common in women than in men (19% vs 11%) [7]. A nasoendoscopy study revealed a difference in velopharyngeal closure between men and women, which could support gender differences in this condition. Men form an acute angle between the soft palate and the posterior pharyngeal wall, whereas women create a right angle, leading to a larger contact area and closure [21].

### 4.3. Etiology

The ultimate common cause of VPI in wind musicians, excluding cases where anatomical irregularities justify the clinical presentation, appears to be the fatigue of the velopalatine sphincter musculature [15,16,18,25,27]. The level of activity of the velum palatine musculature increases as oral air pressure rises following a non-reflex motor plan [36]. Playing wind instruments requires maintaining high intraoral air pressures in an approximate range of 10 to 126 mmHg, 30 times more than during normal conversation [4,5], which increases the likelihood of reaching muscular fatigue and inadequate closure of the velopharyngeal sphincter [7,25,27]. Supporting this hypothesis, an experimental study inducing fatigue through external air pressure on the velum musculature showed that subjects reaching higher fatigue rates were those subjected to higher pressure values (18.4—25.7 mmHg), even leading to exhaustion [37]. In summary, muscular endurance will depend on the level of air pressure and the strength of contraction, measured by the maximum voluntary contraction value, this being the electromyographic value obtained by asking the patient for a maximum muscular contraction [37]. Furthermore, musical techniques such as staccato, vibrato, or circular breathing represent an added difficulty to sphincter closure [31]. It is important to note that, although this etiology is widely recognized and shared in the reviewed literature, there is no study that solidly demonstrates this cause–effect relationship.

The main determinants identified for the variation in oral air pressure are the type of musical instrument and the characteristics of the performed repertoire [38]. On one hand, it has been observed that the instrument requiring greater minimum pressure and a wider normal range of pressures for musical practice is the oboe due to its organological characteristics [7]. On the other hand, intraoral pressure also changes based on the pitch of the emitted sound (higher frequency, higher pressure) and according to dynamics (higher intensity, higher pressure) [38]. Likewise, isolated peaks of maximum pressure have not been linked to the development of VPI [38]. Thus, it is expected that those instrumentalists who play with higher pressures will also be more susceptible to developing VPI due to accumulated muscular fatigue. In fact, this aligns with what was mentioned earlier, that oboists are often the musicians most affected by VPI [27]. This hypothesis does not hold for clarinetists. All wind musicians are exposed to these pressure values throughout their careers for long periods, yet not all develop VPI. Therefore, there is a different individual susceptibility to sphincter fatigue [37]. Hypothetically, it could be thought that those musicians who have VPI establish abnormal management of sound production mechanisms, resulting in an aberrant increase in oral air pressures, a greater need for muscle contraction force, and greater fatigue. This would be expected to occur especially in beginner or training performers, who have fewer technical skills, as we have seen reported [12,14,16,20,28].

### 4.4. Other Associated Factors

A series of factors related to VPI that are considered to exacerbate or precipitate the symptoms have been identified. These data have been obtained through purely descriptive methods, so their actual association with VPI has not been proven. Likewise, the perception of these factors varies depending on whether they are reported by affected patients, healthcare professionals, or teaching staff (Table 2).

The main otorhinolaryngological antecedents and anatomical anomalies described include a history of previous tonsillectomy, adenoidectomy, and cleft palate with mucous and submucous involvement [4,12,13,15,17,21,22,28]. Particularly, there is a report of a patient with VACTERL syndrome [23], an association of congenital malformations including vertebral defects, anal atresia, renal anomalies, limb abnormalities, and tracheoesophageal fistula. This patient did not show evidence of tracheoesophageal fistula or clefts. Surgical procedures that modify the normal anatomy of the velopalatine sphincter or surrounding structures can result in velopalatine insufficiency by compromising the proper sealing of the sphincter musculature. Patients undergoing surgery in the Waldeyer’s ring area typically present an increase in the cross-sectional area of the nasopharyngeal airway, which can normalize within a period of 3 to 6 months or permanently cause VPI, especially in patients with greater pharyngeal depth. Moreover, irregularities in adenoid tissue in the surgical bed can create channels through which air can inadvertently escape to the nose [39]. It is necessary to individualize each case, weighing the balance between benefit and risk in the indication of nasopharyngeal surgery in wind instrument players due to the professional consequences that may arise from it.

Another mentioned factor is diseases of the upper airway [7,18,25]. Acute infections, such as pharyngitis or tonsillitis, could lead to a local inflammatory environment and mucosal irritation contributing to inadequate sphincter closure. Proper body posture in wind instrument players is essential for artistic performance and for preventing frequent musculoskeletal injuries [25,27]. It has been observed, through magnetic resonance imaging, that a greater cervical extension from a neutral head position increases the total volume of the upper airway, while hyperflexion decreases it [40]. In wind instrument players, these volume changes in the airway resulting from differences in head position could contribute to partially obstructing the optimal airflow. Consequently, it would be necessary to increase oral pressure to overcome that resistance and produce sound. The common endpoint is fatigue of the sphincter musculature. Stage anxiety [7,18,21,25,27] has been associated with an increase in VPI symptoms, probably due to the feeling of lack of control, the state of nervousness, and the increased muscular tension experienced by musicians who suffer from stage fright during performances.

The mouthpiece in brass wind instruments is a cup-shaped metal piece with unchanging acoustic characteristics. However, in the woodwind family, except for the flute, which produces sound through an embouchure, reeds are used. Typically made of wood, from the species *Arundo donax*, their use is limited over time due to the wear their components undergo [41]. The facts that wood is a material subject to the variability of each plant’s nature, that reeds are often crafted by hand, and that is a changing element of the instrument add a series of variables to its final characteristics. Thus, the properties of the wood (e.g., hardness, flexibility, distance between fibers), the caliber, the length, and variations in the structure of each reed derived from manufacturing will determine the resistance to the airflow, conditioning the required level of oral pressure [41]. The mute is a mechanism used to reduce the volume of brass wind instruments and modify their tone. Its use could require an increase in oral airflow pressure, as it leads to an increase in acoustic impedance at different sound frequencies due to resonance phenomena [42]. It has been generally described that the characteristics of the music being played can influence the appearance of VPI symptoms [25], specifically parameters of high registers [15,17,24,27], extreme intensity (*pp* or *ff*) [17,25], extended duration of figurations [15,17], and the use of extended techniques [24,25].

Finally, the presence of symptoms that are reported in some cases upon resuming practice after vacation periods [7,16,18] and with excessive practice hours [18,20,21,23,27] could speak to the role of muscular strength and training in the closure of the velopharyngeal sphincter. The lack of preparation of the velopharyngeal musculature resulting from vacation periods without playing could lead to faulty sphincter closure due to a lack of muscular hypertrophy, decreased fiber recruitment, and greater susceptibility to fatigue. Likewise, exposing the velopharyngeal musculature to increased activity with abrupt increases in practice time could also lead to a higher rate of muscle fatigue due to lack of conditioning [43].

### 4.5. Diagnostic

There is no consensus on the diagnostic strategy for VPI in wind musicians [27]. Despite the frequency of this disorder among musicians, there is a lack of awareness among healthcare professionals regarding this issue. Malick et al. observed that 45.3% of participating physicians were aware of the existence of VPI in wind musicians, and only 26.9% had encountered patients with these characteristics [17]. In general, the use of medical history, physical examination, and nasopharyngoscopy during musical practice are the essential components of the initial evaluation of VPI in wind musicians. Detailed anamnesis and the history of pharyngeal surgery and anatomical alterations are of vital importance. Additionally, other complementary tests are reported, such as speech assessment [3,4,12], spirometry [3], videofluoroscopy [4,12,14,22,24], measurement of oral airflow pressure [4,12,19], electromyography [12], nasality index, spirometry, physical evaluation of posture and muscle tension, and assessment of the instrument [25].

The measurement of nasal pressure using a cannula and a transducer has been proposed as a diagnostic method. If positive air pressures are detected during instrumental practice, a more extensive evaluation for the diagnosis of VPI should be conducted [19]. The benefits that this measurement can offer in aiding diagnosis are questionable, as it does not appear to provide any advantage over medical history and physical examination. However, it may have a more practical application in monitoring the severity of symptoms and assessing the success of treatment. The primary reason a musician with VPI may seek medical consultation is the concern that their symptoms may significantly impact their profession and, therefore, their quality of life. A diagnostic strategy that would help assess the importance of the consequences of this issue and determine which patients would benefit most from treatment would involve conducting quality-of-life impact tests such as Velopharyngeal Insufficiency Effects on Life Outcomes (VELO) [25]. It would be necessary to validate this for this specific group of patients. Teachers play a crucial role not only in detecting VPI symptoms but also in identifying aggravating factors and addressing them, given their close contact with students through individual lessons and their in-depth knowledge of their strengths and weaknesses in musical practice [25].

### 4.6. Treatment

The therapeutic strategies for VPI are highly diverse (surgical or conservative), vary among professionals (speech therapists, otolaryngologists, plastic surgeons), lack clear indications, and their effectiveness is unproven (Table 3) [17,27].

Non-invasive treatment is generally considered the initial approach. The most commonly used options include rest, understood as complete cessation of instrumental practice, and speech therapy rehabilitation. However, it has been observed that the vast majority of the time these choices are inadequate (dry swallowing [23], sucking [28], producing velar phonemes [15]). There is only one reported case in which, after a month of discontinuing musical performance, the patient could play for at least 30 min without symptoms. Subsequently, after a year of speech therapy, they were able to practice their trumpet for 1.5 h without symptoms [14]. Other reported conservative treatments include the use of low-resistance reeds or mouthpieces [25,27]; the use of a nasal clip in a professional trumpeter, which allowed him to play without nasal air leakage for extended periods after previously being unable to do so for more than 45 min [26]; and the use of biofeedback to correct nasal air leaks before producing sound in a group of trombonists [19]. In these cases, the long-term outcomes of these patients are unknown.

Invasive treatment is based on various surgical techniques aimed at remodeling the pharynx. Submucosal injection of different substances (Teflon [13], autologous fat [15,23], hydroxyapatite [20], or hyaluronic acid [24]) results in an increased volume that improves closure tightness. This technique offers rapid recovery, minimal complications, and appears to provide good short- and medium-term results. A more aggressive approach involves performing a pharyngeal flap procedure, with different modifications of the technique [4,16,28], which seems to achieve favorable long-term outcomes with minimal reported complications, such as the formation of aberrant scar tissue [16].

### 4.7. Evidence Limitations

This review highlights relevant limitations presented in the selected studies (Table 1 and Supplementary Table S2), including the low number of patients, an important heterogeneity across studies in the inclusion criteria and design, the lack of control group, and the lack of standardized objective testing of VPI.

## 5. Conclusions

VPI appears to affect a significant portion of wind musicians, causing pharyngeal noises and nasal air emissions that interfere with normal musical practice. Its etiology has not yet been fully demonstrated. Preliminary data from the literature attribute the main cause of fatigue of the velopharyngeal sphincter muscles and all contributing factors that may exacerbate it. Diagnostic and therapeutic management has relied on medical history and otorhinolaryngological physical examination. There is currently no consensus on the best treatment approach, and it is common to initiate therapies in an escalating manner, starting with conservative approaches. It is necessary to implement the use of objective tools to evaluate the outcomes of different treatments used in order to design a patient care protocol focused on risk prevention, diagnosis, and treatment.

## Figures and Tables

**Table 1:** Quality assessment.

Reference	Study Type	Evidence Level	Quality
Weber et al. (1970) [3]	CS	4	Regular
Dibbel et al. (1979) [4]	CS	4	Poor
Shanks, J.C. (1990) [12]	CS	4	Regular
Gordon et al. (1994) [13]	CS	4	Regular
Conley et al. (1995) [14]	CS	4	Regular
Klotz et al. (2001) [15]	CS	4	Poor
McVicar et al. (2002) [16]	CS	4	Regular
Schwab et al. (2004) [7]	D	4	Regular
Malick et al. (2007) [17]	D	4	Regular
Whitehand et al. (2009) [26]	CS	5	Poor
Evans et al. (2011) [18]	D	4	Regular
Visser et al. (2011) [28]	CS	4	Regular
Bennet et al. (2013) [19]	CC	4	Poor
Evans et al. (2014) [27]	D	4	Regular
Raol et al. (2015) [20]	CS	4	Regular
Evans et al. (2015) [21]	CC	4	Poor
Macrae et al. (2015) [22]	CS	4	Regular
Syamal et al. (2017) [23]	CS	4	Poor
Koprowski et al. (2017) [24]	CS	4	Poor
Behel et al. (2021) [25]	SR	4	Poor

Abbreviations: CS: case series, CC: case-control, SR: systematic review, D: descriptive, NA: not applicable, NR: not reported, ND: not determinable.

**Table 2:** Factors related to the presentation of VPI.

Type		Factor	References
Related to the patient’s medical history		Relevant ENT history	[17,27]
		Pharyngeal anatomical anomalies	[27]
		Upper airway disease	[7,18]
		Nasal air escape during speech	[23]
Related to fatigue of the velopharyngeal sphincter	Intrinsic	Stage fright	[7,18,21,25,27]
		Bad corporal posture	[25,27]
	Extrinsic	Sound range	[15,17,24,28]
		Sound intensity	[17,25]
		Long value notes	[15,17]
		Type of repertoire	[21]
		Instrument mouthpiece	[25]
		Use of mute	[25]
		Extended techniques	[24,25]
		Restarting practice after vacation periods	[7,16,18]
		Overloading of practice hours	[18,20,21,23,27]

**Table 3:** Therapeutic options for VPI in wind musicians.

Conservative treatment	Rest, “Wait & see”	[14,17,25,27]
	Speech therapy (suction exercises, velar tensor training, subglottic pressure management, etc.)	[14,15,17,23,25,27,28]
	Maxillary removable prosthesis	[17]
	Nasal clip	[26]
	Biofeedback	[19]
	Instrument modifications	[25,27]
	Guided motor learning	[22]
Invasive treatment	Pharyngeal flap	Superior Base Flap: [4,12,16,17,22] Inferior Base Flap: [28]
	Teflon augmentation injection	[13,17]
	Autologous adipose tissue augmentation injection	[15,23]
	Hydroxyapatite augmentation injection in posterior pharyngeal wall	[20]
	Hyaluronic acid augmentation injection in posterior wall	[24]
	Other approaches	[17]

## Data Availability

Not applicable.

## Supplementary Materials

The following supporting information can be downloaded at: https://www.mdpi.com/article/10.3390/jpm13101477/s1, PRISMA checklist; Table S1: Articles included in the review; Table S2: Extended table of quality assessment; Table S3: Articles excluded in the final review.
