# Supplementary material for: Velopharyngeal Incompetence in Musicians: A State-of-the-Art Review
Source: J Pers Med. 2023 Oct 9;13(10):1477. doi: 10.3390/jpm13101477 (PMC10608608; doi:10.3390/jpm13101477)
Supplement: Supplementary file 1 [file jpm-13-01477-s001.zip › jpm-2588644-supplementary.pdf]

Supplementary Table 1: Articles included in the final review.

Authors (Country, Year)	Study Type	Sample	Risk Factors	Symptoms	Diagnosis	Treatment	Comments
Weber, *et al*. [1] (USA, 1970)	CS	F, 23 yo. Oboe. No relevant clinical history.	NA	- NAE	- Medical History<br>- PE<br>- NPS<br>- Speech Evaluation<br>- X-ray<br>- Cephalometry	NA	- IVP only when playing.<br>- Provides information about diagnostic tests and physiological demands for playing wind instruments.
Dibbel, *et al*. [2] (USA, 1979)	CS	2 cases:<br>- F, 20 yo. Oboe. Submucosal cleft palate.<br>- M, 23 yo. Trumpet. No relevant clinical history.	NA	- NAE<br>- Inability to play.	- Speech Evaluation<br>- Measurement of oral airflow and pressure<br>- Imaging studies	Superior-based V-Y pharyngeal flap	- Successful treatment in both cases.<br>- 2 years after the intervention, asymptomatic and able to continue their careers.
Shanks [3] (USA, 1990)	R-CS	M, 12 yo. Tuba. Adenotonsillectomy	NA	- NAE	- Medical History<br>- PE<br>- Speech evaluation (nasality index)<br>- Measurement of oral air pressure<br>- Videofluoroscopy<br>- EMG (neck extensor)	Superior-based pharyngeal flap	- Careful long-term follow-up.<br>- Utility of surgery in cases refractory to conservative treatment.
Gordon, *et al.* [4] (USA, 1994)	CS	F, 31 yo. Bassoon. Adenotonsillectomy	NA	- NAE<br>- PN	- Medical History<br>- PE<br>- NPS	- Teflon injection	- Safe and precise technique.<br>- Teflon is not the ideal material<br>- The need to research other biocompatible materials.
Conley, *et al.* [5] (USA, 1995)	CS	M, 17 yo. Trumpet. No relevant clinical history.	NA	- NAE<br>- Hypernasality	- Medical History<br>- PE<br>- NPS<br>- Videofluoroscopy	- Cessation of instrumental practice for 1 month.	Importance of a conservative approach for non-professional wind musicians.

				- Nasal regurgitation of liquids		- Pharyngeal muscle training (suction exercises).	
Klotz, *et al.* [6] (USA, 2001)	CS + Review	2 cases:<br>- F, 19 yo. French horn. Cleft palate surgery.<br>- F, 20 yo. Oboe. No relevant clinical history.	- Fatigue (long performances)<br>- High notes<br>- Long notes	- NAE<br>- PN	- Medical History<br>- PE<br>- NPS	- Speech therapy<br>- autologous adipose tissue injection.	- Ambulatory lipoinjection can be a good therapeutic option for small nasal leaks.<br>- The long-term outcome of this technique needs to be further evaluated.
McVicar, *et al.* [7] (USA, 2002)	CS	M, 18 yo.<br>Clarinet.<br>No relevant clinical history.	- Fatigue<br>- Restarting practice after holidays	- NAE<br>- PN<br>- Loss of control of the air column	- Medical History<br>- PE<br>- NPS	- Superior-based pharyngeal flap.	The superior-based sphincter flap should be considered a therapeutic option.
Schwab, *et al.* [8] (Germany, 2004)	Cross-sectional	148 musicians:<br>- 53.5% brass.<br>- 46.4% woodwind.<br><br>*46 out of 148 (31%) musicians with VPI	- Stress<br>- Restarting practice after holidays<br>- Stage fright<br>- Cold<br>- ↑ oral pressure	- NAE	- Measurement of oral pressure (Bronchialis "S" catheter + manometer)<br>- NPS	NA	- Instrumentalists requiring higher oral airflow pressure to initiate a note are more affected.<br>- It is advisable to assess oral airflow pressures in students to prevent VPI.<br>- An anatomical and functional evaluation of each case is recommended to determine the appropriate treatment.
Malick, *et al.* [9] (USA, 2007)	Cross-sectional	156 musicians:<br>- 54% M; 46% F.<br>- 49% brass, 51% woodwind.<br>- Ages 18-39 yo.<br><br>Medical history:<br>- Hearing impairment (7.8%)	- Time of music performed<br>- Sound register<br>- Duration of notes<br>- Sound volume<br>- Type of instrument ($p = 160$)<br>- Relevant medical history ($p > 0.5$)	- NAE<br>- PN	NA	- Speech therapy (47.5%)<br>- Sphincter pharyngoplasty (30%)<br>- Pharyngeal flap (26.8%)<br>- "Wait & see" (19%)<br>- Injection of adipose tissue or Teflon in posterior wall or	- VPI can mark the end of the professional careers of many wind musicians.<br>- There is a lack of information among musicians about the characteristics and management of this issue.

		- Articulation disorder (7.6%)<br>- Cleft palate (3.8%)<br>- Adenoidectomy (17.7%)<br>- Tonsillectomy (13.5%).<br><br>53 out of 156 (34%) musicians with VPI<br><br>160 Doctors:<br>- ENT (37%)<br>- Plastic surgeons (54%)<br>- 86% M; 14% F.				velum of the palate (20%)<br>- Palatal lift (10%)<br>- Removable maxillary prosthesis (4.4%)<br>- Tonsillectomy (2.50%)<br>- Adenoidectomy (1.3%)<br>- Uvulopharyngoplasty (1.9%)<br>- Somnoplasty (0.6%)<br>- Turbinectomy (0.6%)<br>- Rhinectomy (0.6%)	
Whitehand, *et al.* [10] (Australia, 2009)	CS	M, 36 yo.<br>Trumpet.	NA	- NAE	NA	- Nasal clip	- Nasal clip could be a conservative treatment.<br>- More studies are needed
Evans, *et al.* [11] (USA, 2011)	Cross-sectional	77 musicians:<br>- 44% F; 36% M.<br>- Clarinet, oboe, saxophone, bassoon, french horn, and trumpet.<br><br>30 out of 77 (39%) musicians with VPI.	- Muscular fatigue<br>- Stage fright<br>- Study overload<br>- Upper respiratory tract infection<br>- Restarting practice after holidays	- PN (83%)<br>- NAE (17%)<br>- Uncontrolled emission (50%)<br>- Throat strain (33%)<br>- Fatigue (63%)	NA	NA	VPI is a common disorder (39%) among brass and woodwind players.
Visser, *et al.* [12] (The Netherlands, 2011)	CS	2 cases:<br>- F, 18 yo. Clarinet. Insufficient suction during breastfeeding due to nasal regurgitation of liquids.	- Sound register	- PN	- Medical History<br>- PE<br>- NPS	- Speech therapy<br>- Posterior-based pharyngeal flap	The posterior pharyngeal base flap is a feasible therapy with good results.

		- F, 26 yo, Clarinet. Adenotonsillectomy					
Bennet, *et al.* [13] (USA, 2013)	CC	- 6 cases<br>- 4 controls<br><br>No relevant clinical history.<br>Trombone.<br>20 – 25 yo.<br>8 M, 2 F	NA	- NAE<br>- PN<br>- NAE just before the onset of sound	- Medical History<br>- Measurement of nasal pressure (using a nasal cannula)	- Biofeedback	- Measuring nasal air pressure may be a good method to monitor sphincter function during musical practice and to guide biofeedback.<br>- NAE occurred mostly before sound production.
Evans, *et al.* [14] (Australia, 2014)	Cross-sectional	14 respondents:<br>- 7 ENT specialists<br>- 7 speech - therapists.	- ↑oral pressure (79%)<br>- Inadequate muscle closure (67%)<br>- Anatomical abnormalities (64%)<br>- Study overload (64%)<br>- Muscular fatigue (64%)<br>- Psychological predisposition (50%)<br>- Incorrect technique (50%)<br>- Muscular tension (43%)<br>- History of ENT surgery (21%)	- NAE<br>- PN<br>- Fatigue	- Medical History (86%)<br>- NPS (71%)<br>- Speech evaluation (50%)<br>- Measurement of oral pressure during musical practice (50%)<br>- Measurement of airflow/pressure ratio (43%)<br>- Symptom detection (36%)<br>- PE (36%)<br>- Videofluoroscopy (23%)	- "*Wait & see*" (83%)<br>- Instrument modifications (75%)<br>- Speech therapy (67%)<br>- Subglottic pressure management (67%)<br>- Muscular training of the tensor veli palatini (58%)<br>- Pharyngeal injection (58%)<br>- Pharyngoplasty with flap (42%)	- Medical history, PE and NPS are essential for the assessment.<br>- Disparity of opinion on therapeutic management has been found among specialists.
Raol, *et al.* [15] (USA, 2015)	CS	2 cases:<br>- M, 15 yo. Saxophone and clarinet player.<br>- F, 16 yo. Oboe player.	- Study overload (7-9 hours/day)	- NAE	- Medical History<br>- PE<br>- NPS<br>- Nasometry	- Sphincter pharyngoplasty.<br>- Injection of hyaluronic acid in posterior pharyngeal wall.<br>- NS 53/40; NS 43/29	PE and NPS during musical exercise are essential to assess and localise nasal air leakage and to tailor treatment to be effective.

Evans, *et al.* [16] (USA, 2015)	CC	- 8 cases (18 – 35 yo)<br>- 5 controls (19 – 33 yo)<br><br>1 tonsillectomy.<br><br>5 saxophones, 6 clarinets, 2 oboes<br><br>4 M, 9 F	- Time of music performed<br>- Type of repertoire<br>- Performance stage environment<br>- Muscular fatigue	- NAE	- Speech nasality assessment (k = 0.71 – 0.78, p = 0.004)<br>- Spirometry (pre and post MEP) CI = 95%<br>- NPS (k = 0.71 – 1.00, p = 0.004)	NA	- A key aspect of VPI may be the contact of the soft palate against an irregular pharynx, resulting from normal anatomical variations.<br>- Fatigue, type of repertoire and stage environment may explain the aggravation and intermittency of the symptomatology.
Macrae, *et al.* [17] (USA, 2015)	CS	F, 20 a.<br>Clarinet.<br>Family history of cleft lip and cleft palate.	NA	- NAE<br>- PN	- Medical History<br>- PE<br>- NPS<br>- Videofluoroscopy (velum length < 2 SD, velum thickness < 3 SD)	- V-Y pharyngeal flap.<br>- Guided motor learning of speech.	- Following corrective surgery in a clarinettist, a perceptible speech disturbance appeared.<br>- Guided motor learning of speech has been able to restore the pre-surgical voice.<br>- Surgery allows the recovery of musical activity
Syamal, *et al.* [18] (USA, 2017)	R-CS + Review	2 cases:<br>- F, 20 yo. Trombone.<br>- M, 20 yo. Saxophone. VACTERL syndrome (without tracheoesophageal fistula)	- F: Study overload (4 - 5 hours/day)<br>- M: Mild VPI during speech	- NAE	- Medical History<br>- NPS	- Speech therapy<br>- Autologous adipose tissue injection.	The results with autologous fat injection can be variable, but it is a minimally invasive and potentially permanent alternative.
Koprowski, et al. [19] (USA, 2017)	CS	F, 18 yo.<br>Clarinet.	- Fatigue<br>- Extended techniques<br>- High and ultra-high register	- NAE	- Medical History<br>- PE<br>- NPS<br>- Fluoroscopy	- Augmentation of posterior pharyngeal wall with hyaluronic acid (1.8 mL of Deflux).	- Videofluoroscopy and NPS are the key diagnostic tests.<br>- Posterior pharyngeal wall augmentation with

							hyaluronic acid appears to be a safe and effective therapeutic option.
Behel, *et al*. [20] (USA, 2021)	Review	NA	Intrinsic:<br>- Stage fright<br>- Upper respiratory tract infection<br>- ↑ oral pressure<br><br>Extrinsic:<br>- Muscular tension<br>- Muscular fatigue<br>- Poor posture<br>- Inadequate air column<br>- High-resistance instrument mouthpiece<br>- Study overload<br>- Extended techniques<br>- Mute<br>- ↑ sound intensity	- NAE<br>- PN<br>- Hypernasality<br>- Decreased voice intensity<br>- Facial grimaces	- NPS<br>- Videofluoroscopy<br>- RMN<br>- Perceptual speech assessment<br>- PE (posture and muscular tension)<br>- Instrument evaluation	- "*Wait & see*"<br>- Speech therapy<br>- Pharyngeal muscle training<br>- Instrument modifications<br>- ENT surgery	In addition to health professionals, music teachers should be a fundamental link in the prevention and detection of VPI in this group in order to offer a multidisciplinary and individualised management to each patient.

Abbreviations: CS, Case Series; NA, not available; R-CS, Retrospective Case Series; CC, Case-control; F, Female; M, Male; NAE, nasal air emissions; PE, Physical Examination; NPS, Nasopharyngoscopy; PN, Pharyngeal Noises; EMG, Electromyography; MEP, Maximum expiratory pressure; SD, Standard Deviation; NS _/_ (*nasalance score* pre-treatment/post-treatment.

Supplementary Table 2. Extended table of Quality assessment.

References	Study type	Evidence level	#1	#2	#3	#4	#5	#6	#7	#8	#9	#10	#11	#12	Quality
Weber, *et al*. (1970) [1]	CS	4	Yes	Yes	NA	NA	Yes	No	NA	NA	Yes	-	-	-	Regular
Dibbel, *et al*. (1979) [2]	CS	4	Yes	Yes	NR	Yes	Yes	No	NA	NA	No	-	-	-	Poor
James C. Shanks (1990) [3]	CS	4	No	Yes	NA	NA	Yes	Yes	NA	NA	Yes	-	-	-	Regular
Gordon, *et al*. (1994) [4]	CS	4	Yes	Yes	NA	NA	Yes	Yes	Yes	NA	Yes	-	-	-	Regular
Conley, *et al*. (1995) [5]	CS	4	Yes	Yes	NA	NA	Yes	Yes	Yes	NA	Yes	-	-	-	Regular
Klotz, *et al*. (2001) [6]	CS	4	Yes	Yes	NA	Yes	Yes	No	Yes	NA	No	-	-	-	Poor
McVicar, *et al*. (2002) [7]	CS	4	Yes	Yes	NA	NA	Yes	No	Yes	NA	Yes	-	-	-	Regular
Schwab, *et al*. (2004) [8]	D	4	Yes	Yes	Yes	NR	ND	Yes	Yes	NA	NA	NA	NA	NA	Regular
Malick, *et al*. (2007) [9]	D	4	Yes	Yes	Yes	NR	ND	Yes	Yes	NA	NA	NA	NA	NA	Regular
Whitehand, *et al*. (2009) [10]	CS	5	No	No	NA	NA	No	No	No	NA	No	-	-	-	Poor
Evans, *et al*. (2011) [11]	D	4	Yes	Yes	Yes	NR	ND	Yes	Yes	NA	NA	NA	NA	NA	Regular
Visser, *et al*. (2011) [12]	CS	4	Yes	Yes	NR	Yes	Yes	No	Yes	NA	Yes	-	-	-	Regular
Bennet, *et al*. (2013) [13]	CC	4	Yes	Yes	No	Yes	No	Yes	No	No	Yes	Yes	NR	No	Poor
Evans, *et al*. (2014) [14]	D	4	Yes	Yes	Yes	NR	ND	Yes	Yes	NA	NA	NA	NA	NA	Regular
Raol, *et al*. (2015) [15]	CS	4	Yes	Yes	NR	Yes	Yes	Yes	Yes	NA	Yes	-	-	-	Regular
Evans, *et al*. (2015) [16]	CC	4	Yes	Yes	No	Yes	Yes	NR	No	No	Yes	Yes	NR	No	Poor
Macrae, *et al*. (2015) [17]	CS	4	Yes	Yes	NA	NA	Yes	Yes	NA	Yes	Yes	-	-	-	Regular
Syamal, *et al*. (2017) [18]	CS	4	Yes	Yes	NR	Yes	Yes	No	No	NA	Yes	-	-	-	Poor
Koprowski, *et al*. (2017) [19]	CS	4	Yes	Yes	NA	NA	Yes	No	No	NA	Yes	-	-	-	Poor
Behel, *et al*. (2021) [20]	SR	4	Yes	No	Yes	Yes	No	Yes	No	No	NA	-	-	-	Poor

Abbreviations: CS (case series), CC (case-control), SR (systematic review), D (descriptive), NA (not applicable), NR (not reported), ND (not determinable).

#1 CS and D: Was the research question or study objective clearly stated? | CC: Were the research question or objective clearly stated and appropriate? | SR: Is the review based on a focused question adequately described?

#2 CS: Was the study population clearly and completely described, including a case definition? | CC and D: Was the study population clearly specified? | SR: Were the eligibility criteria for included and excluded studies pre-defined and specified?

#3 CS: Were the cases consecutive? | CC: Did the authors justify the sample size? | SR: Did the bibliographic search strategy use a comprehensive systematic approach? | D: Were the study participants representative of those eligible for intervention in the general population?

#4 CS: Were the subjects comparable? | CC: Were controls selected or recruited from the same or similar population that gave rise to the cases? | SR: Were titles, abstracts, and full-text articles reviewed in a dual and independent manner for inclusion and exclusion to minimize bias? | D: Were all eligible participants who met the pre-specified entry criteria enrolled?

#5 CS: Was the intervention clearly described? | CC: Were definitions, inclusion and exclusion criteria, algorithms, or processes used for the selection of cases and controls valid, reliable, and consistently implemented in all participants? | SR: Was the quality of each included study independently rated by two or more reviewers using a standard method to assess its internal validity? | D: Was the sample size large enough to provide confidence in the findings?

#6 CS: Were outcome measures clearly defined, valid, reliable, and consistently implemented in all study participants? | CC: Were cases and controls clearly defined? | SR: Were the included studies listed along with the characteristics and results of each study? | D: Was the intervention clearly described and administered uniformly throughout the study population?

#7 CS: Was the follow-up duration adequate? | CC: Were cases and/or controls randomly selected from eligible participants? | SR: Was publication bias assessed? | D: Were outcome measures pre-specified, defined, reliable, and consistently evaluated among all participants?

#8 CS: Were statistical methods well-described? | CC: Was there the use of concurrent controls? | D: Were outcome assessors blinded to participant interventions? | SR: Was heterogeneity assessed? (Only in meta-analyses).

#9 CS: Were the results well-described? | CC: Could the investigators confirm that the exposure/risk occurred before the development of the condition or event that defined a case? | D: Was the loss to follow-up after baseline 20% or less?

#10 CC: Were exposure/risk measures clearly defined, valid, reliable, and implementable in all participants? | D: Did the statistical methods examine changes in outcome measures from before to after the intervention? | D: Did the statistical methods examine changes in outcome measures from before to after the intervention?

#11 CC: Were exposure/risk assessors blinded to case or control status? | D: Were outcome measures of interest taken multiple times before the intervention and multiple times after it?

#12 CC: Were potential key confounding variables statistically measured in the analysis? | D: If the intervention was carried out at the group level, did the statistical analysis take into account the use of individual-level data to determine group effects?

Supplementary Table 3. Articles excluded in the final review.

Author (country, year)	Study type	Characteristics	Reason for exclusion
Shifman, *et al*. (Israel, 2000) [21]	Cross-sectional	7 patients with neurogenic VPI. Evaluation through speech examination, videonasopharyngoscopy, and videofluoroscopy. Treatment with speech aid prostheses.	Did not include wind musicians
Kahane, *et al*. (USA, 2006) [22]	Cross-sectional	4 bassoonists. 4 M. Evaluation during musical practice using videofluoroscopy and videopharyngoscopy. No symptoms of VPI. No relevant medical history.	The participants did not exhibit symptoms or diagnostic suspicion of VPI.
Evans, *et al*. (Australia, 2010) [23]	Literature review	Review of the pharyngeal musculature function applied to the requirements of wind musicians. Emphasizes the importance of proper functioning of this for adequate velopharyngeal closure.	Non-systematic review of the functional anatomy of the pharyngeal musculature in wind musicians.
William J. Dawson (USA, 2012) [24]	Literature review	Compilation of physical problems related to musical performance in bassoonists based on a literature review and the author's personal experience.	Non-systematic review on medical issues of bassoonists. Not specific to VPI.
Schumacher, *et al*. (Germany, 2013) [25]	Cross-sectional	12 trumpeters. 12 M. Ages 23-30. No clinically relevant medical history. Measurement of pharyngeal muscle motor function through MRI and its implication in the oral and pharyngeal cavity during sound production.	The participants did not exhibit symptoms or diagnostic suspicion of VPI
Bishop, *et al*. (Canada, 2014) [26]	Systematic review (*State-of-the-Art*)	11 studies: 8 report increased pharyngeal arches, velum, and/or pharyngeal wall, 3 allude to isolated increase of posterior pharyngeal wall. Evaluation of velopharyngeal closure defect, volume of injected adipose tissue, assessment tools, follow-up duration, and treatment consequences with autologous adipose tissue injection.	Did not assess the efficacy of autologous adipose tissue injection in wind musicians with VPI.
Kummer, *et al*. (USA, 2015) [27]	Literature review	Information about types, causes, diagnostic methods, and treatment of non-cleft related velopharyngeal dysfunction.	Did not not assess the causes, diagnosis, and treatment of VPI in wind musicians.
Morishita, *et al*. (Japan, 2019) [28]	Case series	1 case. M, clarinetist. Audible nasal escape during musical practice. Nasopharyngoscopy shows air leakage at the adenoid level. Successfully treated with posterior pharyngeal wall lipoinjection.	Japanese language

Abbreviations: M (male), MRI (magnetic resonance imaging), VPI (velopharyngeal insufficiency/incompetence).

REFERENCES:

1. Weber, J.; Chase, R.A. Stress Velopharyngeal Incompetence in an Oboe Player. *Cleft Palate J.* **1970**, *7*, 858–861.
2. Dibbell, D.G.; Ewanowski, S.; Carter, W.L. Successful Correction of Velopharyngeal Stress Incompetence in Musicians Playing Wind Instruments. *Plast. Reconstr. Surg.* **1979**, *64*, 662–664.
3. Shanks, J.C. Velopharyngeal Incompetence Manifested Initially in Playing a Musical Instrument. *J. Voice* **1990**, *4*, 169–171, doi:10.1016/S0892-1997(05)80143-2.
4. Gordon, N.A.; Astrachan, D.; Yanagisawa, E. Videoendoscopic Diagnosis and Correction of Velopharyngeal Stress Incompetence in a Bassoonist. *Ann. Otol. Rhinol. Laryngol.* **1994**, *103*, 595–600, doi:10.1177/000348949410300803.
5. Conley, S.F.; Beecher, R.B.; Marks, S. Stress Velopharyngeal Incompetence in an Adolescent Trumpet Player. *Ann. Otol. Rhinol. Laryngol.* **1995**, *104*, 715–717, doi:10.1177/000348949510400909.
6. Klotz, D.A.; Howard, J.; Hengerer, A.S.; Slupchynskj, O. Lipoinjection Augmentation of the Soft Palate for Velopharyngeal Stress Incompetence. *The Laryngoscope* **2001**, *111*, 2157–2161, doi:10.1097/00005537-200112000-00015.
7. McVicar, R.; Edmonds, J.; Kearns, D. Sphincter Pharyngoplasty for Correction of Stress Velopharyngeal Insufficiency. *Otolaryngol.--Head Neck Surg. Off. J. Am. Acad. Otolaryngol.-Head Neck Surg.* **2002**, *127*, 248–250, doi:10.1067/mhn.2002.127382.
8. Schwab, B.; Schultze-Florey, A. Velopharyngeal Insufficiency in Woodwind and Brass Players. *Med. Probl. Perform. Art.* **2004**, *19*, 21–25, doi:10.21091/mppa.2004.1004.
9. Malick, D.; Moon, J.; Canady, J. Stress Velopharyngeal Incompetence: Prevalence, Treatment, and Management Practices. *Cleft Palate-Craniofacial J. Off. Publ. Am. Cleft Palate-Craniofacial Assoc.* **2007**, *44*, 424–433, doi:10.1597/06-176.1.
10. Whitehand, B.; Gates, P. A Nose Peg: A Possible Simple Solution for Musicians with Velopharyngeal Incompetence. *Otolaryngol.--Head Neck Surg. Off. J. Am. Acad. Otolaryngol.-Head Neck Surg.* **2009**, *141*, 148, doi:10.1016/j.otohns.2009.02.001.
11. Evans, A.; Driscoll, T.; Ackermann, B. Prevalence of Velopharyngeal Insufficiency in Woodwind and Brass Students. *Occup. Med. Oxf. Engl.* **2011**, *61*, 480–482, doi:10.1093/occmed/kqr072.
12. Visser, A.; van der Biezen, J.J. Inferior-Based Pharyngeal Flap for Correction of Stress Velopharyngeal Incompetence in Musicians: Case Reports and Review of the Literature. *J. Plast. Reconstr. Aesthetic Surg. JPRAS* **2012**, *65*, 960–962, doi:10.1016/j.bjps.2011.11.049.
13. Bennett, K.; Hoit, J.D. Stress Velopharyngeal Incompetence in Collegiate Trombone Players. *Cleft Palate-Craniofacial J. Off. Publ. Am. Cleft Palate-Craniofacial Assoc.* **2013**, *50*, 388–393, doi:10.1597/11-181.
14. Evans, A.; Driscoll, T.; Ackermann, B. A Delphi Survey on Diagnosis and Management of Stress Velopharyngeal Insufficiency in Wind Musicians. *Int. J. Speech Lang. Pathol.* **2014**, *16*, 445–455, doi:10.3109/17549507.2013.808701.
15. Raol, N.; Diercks, G.; Hersh, C.; Hartnick, C.J. Stress Velopharyngeal Incompetence: Two Case Reports and Options for Diagnosis and Management. *Int. J. Pediatr. Otorhinolaryngol.* **2015**, *79*, 2456–2459, doi:10.1016/j.ijporl.2015.10.013.
16. Evans, A.; Driscoll, T.; Livesey, J.; Fitzsimons, D.; Ackermann, B. Nasendoscopic Evaluation of Stress Velopharyngeal Insufficiency in Wind Musicians: A Pilot Study. *Med. Probl. Perform. Art.* **2015**, *30*, 47–53, doi:10.21091/mppa.2015.1007.
17. Macrae, T.; Stierwalt, J.A.G.; Behel, K.A. A Case Description of Speech Disturbance and Treatment Following Corrective Surgery for Stress Velopharyngeal Incompetence. *Clin. Linguist. Phon.* **2015**, *29*, 826–839, doi:10.3109/02699206.2015.1055006.
18. Syamal, M.N.; Bryson, P.C. Injection Pharyngoplasty With Autologous Fat as Treatment for Stress Velopharyngeal Insufficiency in Brass and Woodwind Musicians. *JAMA Otolaryngol.-- Head Neck Surg.* **2017**, *143*, 142–146, doi:10.1001/jamaoto.2016.1920.
19. Koprowski, S.; VanLue, M.J.; McCormick, M.E. Treatment of Stress Velopharyngeal Incompetence With Injection of Hyaluronic Acid. *Cleft Palate-Craniofacial J. Off. Publ. Am. Cleft Palate-Craniofacial Assoc.* **2018**, *55*, 615–618, doi:10.1177/1055665617732788.
20. Behel, K.; Evans, A.; Rennie-Salonen, B. Synthesizing the Literature on Stress Velopharyngeal Insufficiency in Musicians: Recommendations for Music Educators. *Update Appl. Res. Music Educ.* **2022**, *40*, 27–36, doi:10.1177/87551233211030167.
21. Shifman, A.; Finkelstein, Y.; Nachmani, A.; Ophir, D. Speech-Aid Prostheses for Neurogenic Velopharyngeal Incompetence. *J. Prosthet. Dent.* **2000**, *83*, 99–106, doi:10.1016/s0022-3913(00)70094-1.

22. Kahane, J.C.; Beckford, N.S.; Chorna, L.B.; Teachey, J.C.; McClelland, D.K. Videofluoroscopic and Laryngoscopic Evaluation of the Upper Airway and Larynx of Professional Bassoon Players. *J. Voice Off. J. Voice Found.* **2006**, *20*, 297–307, doi:10.1016/j.jvoice.2005.03.003.
23. Evans, A.; Ackermann, B.; Driscoll, T. Functional Anatomy of the Soft Palate Applied to Wind Playing. *Med. Probl. Perform. Art.* **2010**, *25*, 183–189.
24. Dawson, W.J. Bassoonists' Medical Problems-Current State of Knowledge. *Med. Probl. Perform. Art.* **2012**, *27*, 107–112.
25. Schumacher, M.; Schmoor, C.; Plog, A.; Schwarzwald, R.; Taschner, C.; Echternach, M.; Richter, B.; Spahn, C. Motor Functions in Trumpet Playing-a Real-Time MRI Analysis. *Neuroradiology* **2013**, *55*, 1171–1181, doi:10.1007/s00234-013-1218-x.
26. Bishop, A.; Hong, P.; Bezuhly, M. Autologous Fat Grafting for the Treatment of Velopharyngeal Insufficiency: State of the Art. *J. Plast. Reconstr. Aesthetic Surg. JPRAS* **2014**, *67*, 1–8, doi:10.1016/j.bjps.2013.09.021.
27. Kummer, A.W.; Marshall, J.L.; Wilson, M.M. Non-Cleft Causes of Velopharyngeal Dysfunction: Implications for Treatment. *Int. J. Pediatr. Otorhinolaryngol.* **2015**, *79*, 286–295, doi:10.1016/j.ijporl.2014.12.036.
28. Morishita, D.; Sano, D.; Arai, Y.; Isono, Y.; Tanabe, T.; Inamo, M.; Oridate, N. Lipoinjection Augmentation of the Pharyngeal Posterior Wall for Stress Velopharyngeal Insufficiency/ Incompetence in a Woodwind Musician: A Case Report. *Nippon Jibiinkoka Gakkai Kaiho* **2019**, *123*, 251–256, doi:10.3950/jibiinkoka.123.251.

PRISMA 2020 Main Checklist

Topic	No.	Item	Location where item is reported
**TITLE**			
**Title**	1	Identify the report as a systematic review.	State of the art review with PRISMA search methodology
**ABSTRACT**			
**Abstract**	2	See the PRISMA 2020 for Abstracts checklist	
**INTRODUCTION**			
**Rationale**	3	Describe the rationale for the review in the context of existing knowledge.	introduction
**Objectives**	4	Provide an explicit statement of the objective(s) or question(s) the review addresses.	introduction
**METHODS**			
**Eligibility criteria**	5	Specify the inclusion and exclusion criteria for the review and how studies were grouped for the syntheses.	methodology
**Information sources**	6	Specify all databases, registers, websites, organisations, reference lists and other sources searched or consulted to identify studies. Specify the date when each source was last searched or consulted.	2.5. Search strategy

Topic	No.	Item	Location where item is reported
**Search strategy**	7	Present the full search strategies for all databases, registers and websites, including any filters and limits used.	2.5. Search strategy
**Selection process**	8	Specify the methods used to decide whether a study met the inclusion criteria of the review, including how many reviewers screened each record and each report retrieved, whether they worked independently, and if applicable, details of automation tools used in the process.	2.5. Search strategy
**Data collection process**	9	Specify the methods used to collect data from reports, including how many reviewers collected data from each report, whether they worked independently, any processes for obtaining or confirming data from study investigators, and if applicable, details of automation tools used in the process.	2.5. Search strategy
**Data items**	10a	List and define all outcomes for which data were sought. Specify whether all results that were compatible with each outcome domain in each study were sought (e.g. for all measures, time points, analyses), and if not, the methods used to decide which results to collect.	2.4. Outcomes
	10b	List and define all other variables for which data were sought (e.g. participant and intervention characteristics, funding sources). Describe any assumptions made about any missing or unclear information.	2.1-2.5
**Study risk of bias assessment**	11	Specify the methods used to assess risk of bias in the included studies, including details of the tool(s) used, how many reviewers assessed each study and whether they worked independently, and if applicable, details of automation tools used in the process.	2.6
**Effect measures**	12	Specify for each outcome the effect measure(s) (e.g. risk ratio, mean difference) used in the synthesis or presentation of results.	N/A
**Synthesis methods**	13a	Describe the processes used to decide which studies were eligible for each synthesis (e.g. tabulating the study intervention characteristics and comparing against the planned groups for each synthesis (item 5)).	N/A

Topic	No.	Item	Location where item is reported
	13b	Describe any methods required to prepare the data for presentation or synthesis, such as handling of missing summary statistics, or data conversions.	N/A
	13c	Describe any methods used to tabulate or visually display results of individual studies and syntheses.	N/A
	13d	Describe any methods used to synthesize results and provide a rationale for the choice(s). If meta-analysis was performed, describe the model(s), method(s) to identify the presence and extent of statistical heterogeneity, and software package(s) used.	N/A
	13e	Describe any methods used to explore possible causes of heterogeneity among study results (e.g. subgroup analysis, meta-regression).	N/A
	13f	Describe any sensitivity analyses conducted to assess robustness of the synthesized results.	N/A
**Reporting bias assessment**	14	Describe any methods used to assess risk of bias due to missing results in a synthesis (arising from reporting biases).	N/A
**Certainty assessment**	15	Describe any methods used to assess certainty (or confidence) in the body of evidence for an outcome.	N/A
**RESULTS**			
**Study selection**	16a	Describe the results of the search and selection process, from the number of records identified in the search to the number of studies included in the review, ideally using a flow diagram.	figure 1
	16b	Cite studies that might appear to meet the inclusion criteria, but which were excluded, and explain why they were excluded.	Table 2
**Study characteristics**	17	Cite each included study and present its characteristics.	Results; supplementary table 1.
**Risk of bias in studies**	18	Present assessments of risk of bias for each included study.	table 1

Topic	No.	Item	Location where item is reported
**Results of individual studies**	19	For all outcomes, present, for each study: (a) summary statistics for each group (where appropriate) and (b) an effect estimate and its precision (e.g. confidence/credible interval), ideally using structured tables or plots.	supplementary table 1.
**Results of syntheses**	20a	For each synthesis, briefly summarise the characteristics and risk of bias among contributing studies.	table 1
	20b	Present results of all statistical syntheses conducted. If meta-analysis was done, present for each the summary estimate and its precision (e.g. confidence/credible interval) and measures of statistical heterogeneity. If comparing groups, describe the direction of the effect.	N/A
	20c	Present results of all investigations of possible causes of heterogeneity among study results.	N/A
	20d	Present results of all sensitivity analyses conducted to assess the robustness of the synthesized results.	N/A
**Reporting biases**	21	Present assessments of risk of bias due to missing results (arising from reporting biases) for each synthesis assessed.	N/A
**Certainty of evidence**	22	Present assessments of certainty (or confidence) in the body of evidence for each outcome assessed.	N/A
**DISCUSSION**			
**Discussion**	23a	Provide a general interpretation of the results in the context of other evidence.	Different sections
	23b	Discuss any limitations of the evidence included in the review.	Limitations section
	23c	Discuss any limitations of the review processes used.	Limitations section
	23d	Discuss implications of the results for practice, policy, and future research.	Conclusion
**OTHER INFORMATION**			
**Registration and protocol**	24a	Provide registration information for the review, including register name and registration number, or state that the review was not registered.	N/A

Topic	No.	Item	Location where item is reported
	24b	Indicate where the review protocol can be accessed, or state that a protocol was not prepared.	N/A
	24c	Describe and explain any amendments to information provided at registration or in the protocol.	N/A
**Support**	25	Describe sources of financial or non-financial support for the review, and the role of the funders or sponsors in the review.	Funding section
**Competing interests**	26	Declare any competing interests of review authors.	No competing interests
**Availability of data, code and other materials**	27	Report which of the following are publicly available and where they can be found: template data collection forms; data extracted from included studies; data used for all analyses; analytic code; any other materials used in the review.	N/A

PRIMSA Abstract Checklist

Topic	No.	Item	Reported?
**TITLE**			
**Title**	1	Identify the report as a systematic review.	No
**BACKGROUND**			
**Objectives**	2	Provide an explicit statement of the main objective(s) or question(s) the review addresses.	Yes
**METHODS**			
**Eligibility criteria**	3	Specify the inclusion and exclusion criteria for the review.	Yes
**Information sources**	4	Specify the information sources (e.g. databases, registers) used to identify studies and the date when each was last searched.	Yes
**Risk of bias**	5	Specify the methods used to assess risk of bias in the included studies.	No
**Synthesis of results**	6	Specify the methods used to present and synthesize results.	No
**RESULTS**			

Topic	No.	Item	Reported?
**Included studies**	7	Give the total number of included studies and participants and summarise relevant characteristics of studies.	Yes
**Synthesis of results**	8	Present results for main outcomes, preferably indicating the number of included studies and participants for each. If meta-analysis was done, report the summary estimate and confidence/credible interval. If comparing groups, indicate the direction of the effect (i.e. which group is favoured).	Yes
**DISCUSSION**			
**Limitations of evidence**	9	Provide a brief summary of the limitations of the evidence included in the review (e.g. study risk of bias, inconsistency and imprecision).	Yes
**Interpretation**	10	Provide a general interpretation of the results and important implications.	Yes
**OTHER**			
**Funding**	11	Specify the primary source of funding for the review.	Yes
**Registration**	12	Provide the register name and registration number.	No

From: Page MJ, McKenzie JE, Bossuyt PM, Boutron I, Hoffmann TC, Mulrow CD, et al. The PRISMA 2020 statement: an updated guideline for reporting systematic reviews. MetaArXiv. 2020, September 14. DOI: 10.31222/osf.io/v7gm2. For more information, visit: www.prisma-statement.org
